# Impact of fermented organic formulations combined with inorganic fertilizers on broccoli (*Brassica oleracea* L. var. *italica* Plenck) cv. Palam Samridhi

**DOI:** 10.1016/j.heliyon.2023.e20321

**Published:** 2023-09-20

**Authors:** Shraddha, Y.R. Shukla, Kuldeep Thakur, Rohit Kumar Vashishat, Subhash Sharma, Rajeshwar Singh Chandel, Sunil Dhingra, Tabish Alam, Rohit Khargotra, Kumari Jyoti

**Affiliations:** a Department of Vegetable Science, College of Horticulture, Dr. YS Parmar University of Horticulture and Forestry, Nauni, Solan, Himachal Pradesh, India; b Department of Social Sciences, College of Forestry, Dr. YS Parmar University of Horticulture and Forestry, Nauni, Solan, Himachal Pradesh, India; c Department of Entomology, College of Horticulture, Dr. YS Parmar University of Horticulture and Forestry, Nauni, Solan, Himachal Pradesh, India; d Department of Mechanical Engineering, U.I.E.T., Kurukshetra University, 136119, India; e Architecture, Planning and Energy Efficiency Group, CSIR-Central Building Research Institute, Roorkee, 247667, India; f Institute of Materials Engineering, Faculty of Engineering, University of Pannonia, Veszprem, 8200, Hungary; g Department of Biotechnology, Govind Ballabh Pant Engineering College, Pauri, Garhwal, Uttarakhand, 246194, India

**Keywords:** Broccoli, Soil fertility, Nutrient uptake, Beejamrit, Jeevamrit, Ghanjeevamrit

## Abstract

A two-year field experiment (2018–19 and 2019–20) was laid out in a randomized complete block design (RCBD) with a spacing of 60 × 45 cm involving three replications with ten treatments having cow manurial amendments along with integrated nutrient management in a plot size of 3.0 m × 1.8 m. The effect of the integration of cow manurial amendments and mineral fertilizers on soil fertility, nutrient uptake, yield, and economics of broccoli was studied. The experiment was laid out during the rabi season in the mid-hills of Himachal Pradesh. T_8_ [90% RDN (112.5 N: 67.5 P: 46.8 K kg/ha) + 5% jeevamrit (1.5 l/m2) + 5% jeevamrit foliar spray] obtained the greatest organic carbon (20.93 g kg^−1^), available N (375.13 kg ha^−1^), P (48.46 kg ha^−1^), K (260.53 kg ha^−1^) in the soil as well as more uptake of N (60.58 kg ha^−1^), P (7.25 kg ha^−1^) and K (37.88 g ha^−1^) by the plants. Further, this treatment obtained the greatest value for yield (186.77 q ha^−1^ and 12.44 kg plot^−1^), net income (₹ 245840) and cost-benefit ratio (1.93). Outcomes of this investigation suggested that combined usage of cow manure, jeevamrit, beejamrit, and ghanjeevamrit with inorganic fertilizers proved to be useful for enhancing soil health, increasing nutrient uptake, and ensuring sustainable production of broccoli.

## 1 Introduction

Broccoli (*Brassica oleracea* L. var. *italica* Plenck, 2n = 2x = 18) is an important exotic vegetable belonging to the Brassicaceae family. It is a native of Eastern Mediterranean region with Italy being the main centre of diversity. Vitamin A content in Broccoli is 3500-IU which is 130 times higher than that of cauliflower and 22 times more than cabbage [1]. In Himachal Pradesh, it is one of the most significant vegetable crops of mid and high hills. The produce is marketed in the north Indian plains as an off-season vegetable. After the “green revolution”, there was increase in vegetables production due to uncontrolled use of synthetic fertilizers, but their large-scale use has resulted in soil heath depletion, ecological hazards and exhaustion of non-renewable sources of energy. Due to awareness regarding soil health and excessive use of chemical fertilizers in modern day farming, there was a shift from conventional methods to integrated nutrient management systems [2]. With the use of synthetic fertilizers, nitrate accumulation takes place in broccoli which can have negative impact on human health. To reduce these nitrate accumulates, organic manures can be used as substitutes of synthetic fertilizers [3]. Chemical or synthetic fertilizers can be replaced by organic manures [4,5] which makes a contribution in improving soil structure [6,7] and microbial biomass [8]. The components used to prepare liquid organic fermented manures like jeevamrit, beejamrit and ghanjeevamrit are easily available in farm *viz.* cow dung, urine, milk, curd, ghee, legume flour and jaggery. There is presence of essential macronutrients, micro nutrients, many vitamins, essential amino acids, beneficial microorganisms and plant growth regulators like Indole Acetic Acid, Gibberellic Acid in these preparations [9,10]. As a result, yield is increased and farmers get maximum benefit. In addition to easily available nutrients, fermented liquid manures also contain plant growth stimulants and a larger microbial load, which aid in promoting plant growth, metabolic activity, and disease, pest, and insect resistance. As a result, plants yield more and productivity is increased [11,12].

## 2 Material and methods

The investigation was carried out in the Department of Vegetable Science, Experimental Research Farm, Dr YS Parmar University of Horticulture and Forestry, Nauni, Solan, Himachal Pradesh during rabi seasons of 2018–19 and 2019–20. Field experiment was conducted having three replications and was laid out in Randomised Complete Block Design (RCBD). The data recorded during the experiment was statistically analyzed by using the Microsoft-Excel and OPSTAT and SPSS 16.0 packages. Analysis of variance was conducted according to RCBD on the combined 2-year data set. A combination of fermented organic manures and synthetic fertilizers were in the treatments and treatment combinations are given in Table 1. Approximately one month old seedling of broccoli cv. “Palam Samridhi” were transplanted on 8^th^ October 2018 and 14^th^ October 2019 with the plot size of 3.0 m × 1.8 m and a spacing of 60 × 45 cm consisting of 20 plants. Calculations were done on the basis of the treatments for farm-yard manure (FYM), urea, single super phosphate (SSP) and muriate of potash (MOP) and desired quantities were incorporated to the soil by broadcasting method in individual plots. Split dosage of urea was applied; first half before transplanting and the other half in two splits, first one month of transplanting (seedling stage) and remaining after 1 month of first application (late vegetative stage/early flowering).

**Table 1:** Details of the treatments used in the study.

Treatment Code	Treatment Details
T_1_	RDN {(125 N: 75P:52 K kg/ha) + FYM 20 t/ha}
T_2_	T_1_ + Bj
T_3_	90% RDN + 5% Jv (0.5l/m^2^)
T_4_	90% RDN + 5% Jv (1.0l/m^2^)
T_5_	90% RDN + 5% Jv (1.5l/m^2^)
T_6_	90% RDN + 5% Jv (0.5l/m^2^) + 5% Jv. FS
T_7_	90% RDN +5% Jv (1.0l/m^2^) + 5% Jv. FS
T_8_	90% RDN +5% Jv (1.5l/m^2^) + 5% Jv. FS
T_9_	90% RDN + G. Jv @ 200 kg/ha
T_10_	90% RDN + G. Jv @ 200 kg/ha +5% Jv. FS

Note. T: Treatment; FYM: Farm-yard manure; RDN: Recommended dose of Nutrients; Bj: Seed treatment with Beejamrit; Jv: Jeevamrit soil drenching at fortnightly interval; Jv. FS: Jeevamrit foliar spray starting at 20 days after planting at 20 days interval; G. Jv: Ghanjeevamrit soil application at 15 and 45 days after planting; N: Nitrogen; P: Phosphorus; K: Potassium; kg: Kilogram; ha: hectare; L: litre; m^2^: square meters.

### 2.1 Preparation of fermented organic manures

**Fig. 1:**
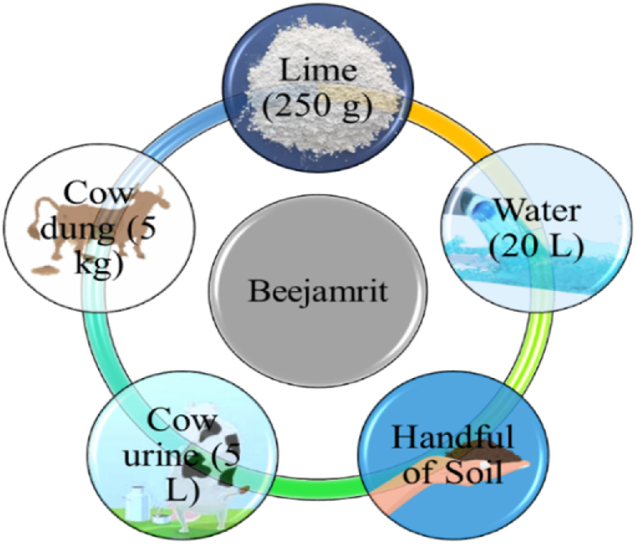
Elements used for preparation of beejamrit.

Beejamrit: Mixed the ingredients (shown in Fig. 1), then left outside for 24 h to allow the fermentation process. The mixture was mixed twice a day. The seeds were then dipped in this concoction and left to dry in the shade.

**Fig. 2:**
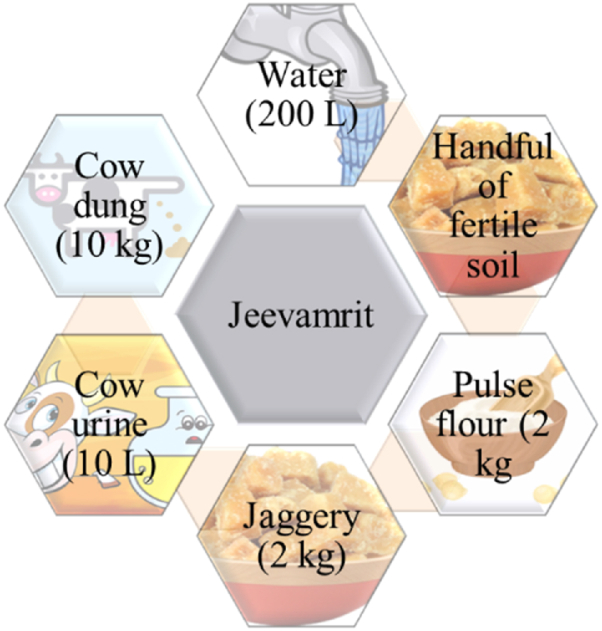
Elements used for preparation of jeevamrit.

Jeevamrit: Mixed the ingredients and stir the mixture twice a day, in a clockwise manner. On the fifth day, the solution was filtered, and the filtrate was diluted for soil drenching. Jeevamrit @ 5% (5 l per 100 l of water) was administered to the soil at fortnightly intervals. The first treatment was made on the seventh day after sowing, and the final application was made fifteen days prior to harvest. The filtrate was diluted with water for foliar application, and a 5% solution was sprayed every 20 days. The ingredients are shown in Fig. 2.

**Fig. 3:**
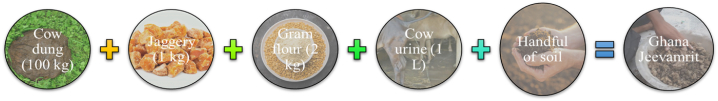
Elements used for preparation of ghana jeevamrit.

Ghana Jeevamrit: All ingredients (shown in Fig. 3.) were combined and made into a paste. Fermentation was allowed for two days while being covered with a gunny bag. Later, the mixture was shaped into a walnut-sized ball and put in the field close to the plant's roots at 15 and 45 days following transplanting.

The nutrient composition of Beejamrit, Jeevamrit and Ghana Jeevamrit (Table 2) reveals that they are good source of nutrients.

**Table 2:** Chemical content of the fermented organic formulations.

Input	Nutrient content	
Beejamrit [13,14]	pH	8.2
	EC	5.5 dSm^−1^
	Nitrogen	0.04 g l^−1^
	Phosphorus	0.14 g l^−1^
	Potassium	0.26 g l^−1^
Jeevamrit [15]	pH	7.07
	EC	3.4 dSm^−1^
	Carbon	7.19 g l^−1^
	Nitrogen	2.8 g l^−1^
	Phosphorus	1.7 g l^−1^
	Potassium	2.0 g l^−1^
Ghana Jeevamrit [16,17,18]	Nitrogen	14.7 g kg^−1^
	Phosphorus	12.8 g kg^−1^
	Potassium	9.0 g kg^−1^

Note. kg: Kilogram; g: gram; L: litre; pH: negative logarithm of H^+^; EC: Electrical conductivity; dS m^−1^: Decisiemens per metre; g l^−1^: gram per litre; g kg^−1^: gram per kilogram.

The soil samples were collected from each plot to a depth of 15 cm using soil auger before sowing and after harvesting of the crop. A composite sample was created by combining soil samples from several locations. The excess soil was quartered and 500 g saved for analysis. Mixed the remaining two quarters once again and the procedure was repeated until only 500 g of soil left. To prepare soil samples for chemical analysis, they were ground and passed through a 2 mm screen, and then placed in cotton bags to dry naturally. Soil pH and EC were estimated using a conductivity metre, organic carbon content was estimated using the Walkely-Black method (1934) [19], available N and P in the soil were determined using the alkaline potassium permanganate method [20], and and Olsen’s method [21] available K was determined using the standard neutral ammonium acetate method [22]. Each plot's composite sample of ten plants was selected at random when they were fully mature [23]. Following 0.1 N HCl washing, distilled water was used to rinse all of the samples. The cleaned samples were dried in the air first, and then at 60 °C in the oven until they reached a constant weight. Grinding of dried samples was done in an electric grinder and the powder was stored in butter paper bags for further chemical analysis to be carried out in laboratory. For the N estimation, 1 g of plant sample was digested in concentrated H_2_SO_4_ along with digestion mixture. Following digestion, the N was estimated using the micro-Kjeldahl method. 1 g of the plant sample was digested in a 4:1 mixture of nitric acid and perchloric acid (HNO_3_: HClO_4_) in order to estimate the amounts of P and K. Three washes with distilled water were provided to the digestion flask in order to completely transfer the digestion material, and the volume was increased to 100 ml. Spectronic-20 and the Vanado-molybdate yellow colour method was used to measure phosphorus, while flame photometer was used to determine potassium in the extract. The N, P and K (%) content in plant was then multiplied by its biomass on dry weight basis as shown below for computing nutrient uptake in kg ha^−1^.

(1) $$\text{Biomass}\;\left(\text{kg}/\text{ha}\right)=\frac{\text{Weight}\;\text{of}\;\text{dry}\;\text{plant}\times 10,000}{\text{Area}\;\text{covered}\;\text{by}\;\text{the}\;\text{plant}\times 1000}$$

(2) $$\text{Nutrient}\;\text{Uptake}\;\left(\text{kg}/\text{ha}\right)=\frac{\text{Biomass}\;\left(\text{kg}/\text{ha}\right)\times \text{Nutrient}\;\text{content}\;\left(\%\right)}{100}$$

All of the plants in a plot were measured to obtain recorded data for their central head, secondary head (kg), and yield per hectare was calculated and expressed in quintals. The cost of cultivation was recorded and economics was worked out of broccoli cultivation under different treatments. The yield (q ha^−1^) was multiplied by the sale rate (₹) to work out gross income.

(3) $$\text{Gross}\;\text{income}=\text{Yield}\;\left(q\;{\text{ha}}^{-1}\right)\times \text{sale}\;\text{rate}\;\left(₹\;2,000/q\right)$$

Total cost of cultivation was calculated as the sum of fixed cost per hectare, risk factor, management factor and cost of the treatment per hectare.

(4) Totalcostofcultivation=Fixedcost+Variablecost

The total cost of cultivation was subtracted from gross income to get net income.

(5) Netincome=Grossincome–Totalcostofcultivation

Cost: benefit ratio was obtained by dividing net income by the total cost of cultivation [[11], [24]].

(6) $$\text{Cost}:\text{benefit}\;\text{ratio}=\frac{\text{Net}\;\text{income}}{\text{Total}\;\text{cost}\;\text{of}\;\text{cultivation}}$$

## 3 Result and discussion

Soil organic carbon content from T_8_ (20.93 g kg^−1^) had positive significance over all the treatments while lowest soil organic carbon was obtained from T_1_ (15.1 g kg^−1^) (Table 3). Increased levels of organic carbon content might be attributed to decrease in bulk density which might be due to the application of Jeevamrit [25,13]. Similar results were reported by Saharan and Sharma & Chadak when Jeevamrit and farm yard manure were incorporated in the field, it not only boosted the soil's nutritional content but also influenced the composition of the microbial community, enhanced the soil's structure, water-holding ability, and soil organic carbon [26,18].

**Table 3:** Effect of inorganic and cow manurial amendments on pH, EC, and OC of soil.

Treatment code	pH			EC (dS m^−1^)			OC (g kg^−1^)		
	Years			Years			Years		
	2018–19	2019–20	Pooled	2018–19	2019–20	Pooled	2018–19	2019–20	Pooled
T_1_	6.73	6.70	6.72	0.43	0.44	0.44	12.30	17.90	15.10
T_2_	6.90	6.97	6.93	0.46	0.45	0.45	12.47	18.43	15.45
T_3_	6.87	6.97	6.92	0.46	0.47	0.46	14.33	21.43	17.88
T_4_	6.77	6.73	6.75	0.42	0.44	0.43	14.50	20.07	17.28
T_5_	6.80	6.77	6.78	0.45	0.45	0.45	14.47	24.30	19.38
T_6_	6.83	6.80	6.82	0.43	0.44	0.44	15.03	22.90	18.97
T_7_	6.93	7.03	6.98	0.45	0.46	0.46	15.43	24.70	20.07
T_8_	7.10	7.07	7.08	0.47	0.49	0.48	16.10	25.77	20.93
T_9_	6.80	6.77	6.78	0.44	0.45	0.45	13.60	19.13	16.37
T_10_	6.93	7.03	6.98	0.44	0.45	0.45	13.99	19.87	16.93
Mean	6.87	6.88	6.88	0.45	0.45	0.45	14.22	21.45	17.84
CD _(0.05)_	NS	NS		NS	NS		1.02	1.38	
Y									0.38
T									0.85
Y × T									1.21

Note. T: Treatment; pH: negative logarithm of H^+^ (concentration power of hydrogen); EC: Electrical conductivity is a measurement of the quantity of soluble salts in soil as well as the soil's capacity to conduct electrical current; dS m^−1^: Decisiemens per metre; OC: A quantifiable part of soil organic matter is called soil organic carbon; g kg^−1^: gram per kilogram of soil; CD: Critical difference; Y: Years; NS: Non-significant.

Values of available N (326.91 kg ha^−1^ and 329.14 kg ha^−1^), P (40.25 kg ha^−1^ and 42 kg ha^−1^) and K (215.85 kg ha^−1^ and 222.8 kg ha^−1^) content before planting of broccoli were recorded for both the years 2018–19 and 2019–20 respectively. Available nutrients (Table 4) were more in T_8_ [available N (375.13 kg ha^−1^), P (48.46 kg ha^−1^), K (260.53 kg ha^−1^)]. This available nutrient content in soil could result from the application of fermented liquid organic manures *viz.* Jeevamrit and Beejamrit which might have boosted up the growth of nitrogen and phosphorus solubilizing bacteria in soil. The availability of available NPK content in soil is due to supplementation of soil with the organic manures which greatly enhanced basal respiration due to presence of great number of microorganisms [27]. Availability of potassium could be because of the presence of organic manures and reduced fixation of potassium. With the increase in organic matter, it might have interacted with the clay complex of potassium in order to transfer potassium from the non-exchangeable portion to the soil's available pool [9,28,29].

**Table 4:** Effect of inorganic and cow manurial amendments on available NPK of soil.

Treatment Code	N (kg ha^−1^)			P (kg ha^−1^)			K (kg ha^−1^)		
	Years			Years			Years		
	2018–19	2019–20	Pooled	2018–19	2019–20	Pooled	2018–19	2019–20	Pooled
T_1_	240.14	244.20	242.17	36.28	38.17	37.23	209.66	210.68	210.17
T_2_	247.69	259.71	253.70	36.51	38.68	37.59	215.00	215.73	215.37
T_3_	300.91	306.28	303.60	40.58	43.38	41.98	241.11	243.98	242.55
T_4_	302.61	309.15	305.88	38.78	41.11	39.95	230.93	233.14	232.03
T_5_	323.12	327.43	325.28	41.13	44.18	42.66	245.01	246.95	245.98
T_6_	342.73	350.70	346.72	42.21	45.14	43.67	247.19	250.66	248.93
T_7_	366.29	370.89	368.59	45.55	48.84	47.19	254.72	256.36	255.54
T_8_	373.11	377.15	375.13	46.86	50.05	48.46	261.51	259.55	260.53
T_9_	271.72	275.96	273.84	37.35	40.39	38.87	219.95	220.98	220.46
T_10_	277.51	278.38	277.95	38.33	40.59	39.46	222.42	225.92	224.17
Mean	304.58	309.99	307.28	40.36	43.05	41.71	234.75	236.39	235.57
CD _(0.05)_	13.32	14.66		1.98	2.34		7.83	6.43	
Y			4.43			0.68			NS
T			9.90			1.52			5.13
Y × T			NS			NS			NS

Note. T: Treatment; CD: Critical difference; Y: Years; NS: Non-significant; N: Nitrogen; P: Phosphorus; K: Potassium; kg ha^−1^: kilogram per hectare.

The nutrient uptake content in broccoli is presented in Table 5 and clearly shows that highest uptake was obtained by T_8_ [N uptake 60.58 kg ha^−1^, P uptake 7.25 kg ha^−1^ and K uptake 37.88 kg ha^−1^]. This might be attributed the increase in nutrient use efficiency which further increased nutrient uptake and yield of the crop by of organic and inorganic fertilizers [30,18]. Both Jeevamrut and Beejamrit exhibited high levels of microbial activity and growth hormones, which may have boosted soil biomass and promoted the availability and absorption of both added and naturally occurring nutrients, resulting in the highest crop growth and production [31]. The increase in nitrogen uptake was noticed with the application of jeevamrit which contains cow urine which is rich in uric acid, a rich source of nitrogen [32]. Foliar spray application of jeevamrit increases the supply of nutrients content and makes the nutrients readily available to the plants. Spehia [33] also showed in Indian palak that foliar spray of 5% jeevamrit along with nutrient film technique had highest nitrogen, phosphorus and potassium uptake because of higher amount of microbes present that encourages metabolic activities.

**Table 5:** Effect of inorganic and cow manurial amendments on NPK uptake by broccoli plants.

Treatment Code	N Uptake (kg ha^−1^)			P Uptake (kg ha^−1^)			K Uptake (kg ha^−1^)		
	**Years**			**Years**			**Years**		
	**2018–19**	**2019–20**	**Pooled**	**2018–19**	**2019–20**	**Pooled**	**2018–19**	**2019–20**	**Pooled**
T_1_	34.30	35.52	34.91	2.53	2.51	2.52	12.60	13.30	12.95
T_2_	36.93	36.28	36.60	3.27	3.70	3.48	13.10	15.56	14.33
T_3_	45.48	45.86	45.67	5.03	5.26	5.15	27.61	25.24	26.42
T_4_	46.51	46.26	46.39	5.17	5.19	5.18	28.53	27.40	27.97
T_5_	52.89	53.55	53.22	5.35	5.70	5.53	32.50	33.12	32.81
T_6_	55.06	55.69	55.37	5.77	5.86	5.82	35.48	35.75	35.61
T_7_	56.40	57.11	56.75	7.13	6.82	6.98	35.77	39.69	37.73
T_8_	60.70	60.46	60.58	7.32	7.17	7.25	39.33	36.43	37.88
T_9_	42.92	43.00	42.96	4.07	3.97	4.02	22.30	23.17	22.73
T_10_	43.83	44.27	44.05	4.60	4.83	4.72	27.09	27.55	27.32
Mean	47.50	47.80	47.65	5.02	5.10	5.06	27.43	27.72	27.58
CD _(0.05)_	6.87	6.78		1.22	1.36		5.05	5.68	
Y			NS			NS			NS
T			4.74			0.90			3.72
Y × T			NS			NS			NS

Note. T: Treatment; CD: Critical difference; Y: Years; NS: Non-significant; N: Nitrogen; P: Phosphorus; K: Potassium; kg ha^−1^: kilogram per hectare.

Integrated use of cow manurial amendments and inorganic fertilizers were found to have significant effect on yield. During 2018–19, treatment T_8_ produced significantly higher yield (12.44 kg plot^−1^ and 182.32 q ha^−1^). Pooled analysis of data revealed that maximum (12.61 kg plot^−1^ and 186.77 q ha^−1^) marketable yield was observed in the same treatment *i.e.*, T_8_ (Table 6). In the present findings, more yields in T_8_ might be due to addition of NPK fertilizers with organics which increased the activity of auxins and microbial saprophytes [34]. Dash [35] observed that with the application of organic manures in broccoli, there might be improvement in the soil properties which ultimately helped in preferable absorption of nutrients and thus more yield. According to Chandrakala [36], inorganic fertilizers might have increased supply of nutrients in the soil at earlier crop growth stages and at later stages, it might have released native soil nutrients. They believed with the presence of IAA, GA_3_ and other nutrients in fermented liquid manures (jeevamrit, beejamurth and panchagavya), there might be an increased number of fruits per plant thereby increasing yield. Somasundaram [37] emphasised that foliar spray of these organic manures along with soil application acted as a stimulus which further increased growth regulator production in the cell system. According to Boraiah [38], yield always depends upon the assimilatory surface of the plant in terms of vegetative characters *viz.* plant height, leaf area index, number of branches and leaves. All these growth characters noted increased vigor in capsicum with the use of jeevamrit and as a result, distribution of assimilates to different crop parts increased which ultimately increased the yield.

**Table 6:** Effect of inorganic and cow manurial amendments on marketable yield of broccoli.

Treatment Code	Marketable yield per plot (kg)			Marketable yield per ha (q)		
	Years			Years		
	2018–19	2019–20	Pooled	2018–19	2019–20	Pooled
T_1_	9.95	10.46	10.21	147.47	154.91	151.19
T_2_	10.03	10.53	10.28	148.61	156.02	152.32
T_3_	10.33	10.91	10.62	153.07	161.61	157.34
T_4_	10.99	11.19	11.09	162.81	165.80	164.30
T_5_	11.39	11.72	11.55	168.67	173.56	171.11
T_6_	11.56	11.77	11.67	171.33	174.43	172.88
T_7_	11.77	12.44	12.10	174.36	184.27	179.32
T_8_	12.44	12.77	12.61	184.32	189.21	186.77
T_9_	10.12	10.62	10.37	149.86	157.33	153.59
T_10_	10.44	10.69	10.56	154.63	158.35	156.49
Mean	10.90	11.31	11.11	161.51	167.55	164.53
CD_(0.05)_	0.61	0.59		9.05	8.78	
Y			0.18			2.70
T			0.41			6.04
Y × T			NS			NS

Note. T: Treatment; CD: Critical difference; Y: Years; NS: Non-significant; kg: kilogram; ha: hectare; q: quintals.

The observations computed on economics of integrated use of cow manurial amendments and inorganic fertilizers have been presented in Table 7. Pooled data showed that the maximum net income of ₹ 2,45,840 was obtained in treatment T_8_ followed by T_7_ (90% of recommended dose of fertilizers + 5% drenching with jeevamrit @ 1.0 L m^−2^ + 5% foliar spray with jeevamrit) with net income of ₹ 2,34,757. According to Ola [39], the highest benefit cost ratio might be due to changes in the form of plant growth, yield and quality parameters. This improvement led to the proportional increase in yield and higher B:C ratio. The results of Chandrakala [25] disclosed that combined application of jeevamrit, beejamrit, panchagavya and ghanjeevamrit had maximum gross income and net worth with lower cost of cultivation because these organic manures were produced on the farm without any additional cost. Manjunatha [40] were of the opinion that the lowest benefit cost ratio of the treatment having recommended dosage of manures might be due to high cost of the fertilizers. According to them, application of jeevamrit was economically beneficial as it aggravated the activity of microbes in soil thereby solubilizing nutrients in the soil resulting in higher uptake and increased productivity in sunflower.

**Table 7:** Effect of inorganic and cow manurial amendments on economics.

Treatment code	Yield (q/ha)	Cost of Cultivation (₹/ha)	Gross Income (₹/ha)	Net Income (₹/ha)	B:C Ratio
T_1_	151.19	111573	302383	190810	1.71
T_2_	152.32	112023	304637	192613	1.72
T_3_	157.34	113773	314677	200903	1.77
T_4_	164.30	118143	328607	210463	1.78
T_5_	171.11	121963	342227	220263	1.81
T_6_	172.88	119503	345757	226253	1.89
T_7_	179.32	123873	358630	234757	1.90
T_8_	186.77	127693	373533	245840	1.93
T_9_	153.59	108513	307183	198670	1.83
T_10_	156.49	114243	312977	198733	1.74
CD _(0.05)_	6.04				0.11

Note. T: Treatment; CD: Critical difference; q/ha: quintal per hectare; ₹/ha: Indian Rupees per hectare; B:C ratio: Cost benefit ratio.

## 4 Conclusion

Based on the results, the conclusion can be drawn that treatment T_8_ (90% RDN + 5% jeevamrit @ 1.5 l/m^2^ + 5% jeevamrit foliar spray) produced the best outcome in terms of production, yield and B:C ratio in broccoli. Further it increased soil fertility and nutrient uptake by the plant because it contain abundant of microbes which enrich the soil. Therefore, it can be concluded that broccoli can be grown economically and profitably with 90% RDN along with drenching of 5% jeevamrit @ 1.5 l/m^2^ at fort nighty interval in conjunction with 5% jeevamrit as foliar spray at 20 days interval. Major challenges faced during the research trial were regular preparation of fermented organic manures which is time-consuming, and application is difficult.

## Author contribution statement

Rohit Khargotra: Analyzed and interpreted the data; Wrote the paper.

Shraddha Kumari: Conceived and designed the experiments; Performed the experiments.

Y.R Shukla and Subhash Sharma: Conceived and designed the experiments.

Kuldeep Thakur: Analyzed and interpreted the data; Wrote the paper.

Rohit Kumar Vashishat and Rajeshwar Singh Chandel: Contributed the reagents, materials, analysis tools or data.

Sunil Dhingra and Tabish Alam: Analyzed and interpreted the data.

Jyoti Kumari: Contributed the reagents, materials, analysis tools or data; Wrote the paper.

## Data availability statement

Data will be made available on request.

## Additional information

No additional information is available for this paper.

## Declaration of competing interest

The authors declare that they have no known competing financial interests or personal relationships that could have appeared to influence the work reported in this paper.
